# Case report and literature analysis: solitary HCC- recurrence in the right maxillary sinus after curative resection

**DOI:** 10.3389/fonc.2024.1279126

**Published:** 2024-01-29

**Authors:** Tinotenda Blessing Madzikatire, Yunfeng Shan

**Affiliations:** Department of Hepatobiliary Surgery, The First Affiliated Hospital of Wenzhou Medical University, Wenzhou, Zhejiang, China

**Keywords:** solitary, hepatocellular carcinoma, recurrence, metastasis, maxillary sinus mass

## Abstract

**Background:**

The primary treatment for eligible patients with hepatocellular carcinoma (HCC) is curative liver resection, offering a 5-year overall survival (OS) of 60%-80%. Despite this, the recurrence rate within five years post-resection remains notably high, ranging from 40% to 50%. Instances of recurrence in the maxillary sinus following liver resection are exceedingly uncommon. This report details a unique case of solitary maxillary sinus recurrence after the curative resection of HCC, which underwent maxillary tumor resection, along with a comprehensive review of pertinent literature. No similar cases have been documented previously.

**Case presentation:**

In 2014, an 85-year-old male patient was diagnosed with HCC and subsequently underwent left hepatectomy. Following the resection, the patient underwent a 9-year follow-up period without any evidence of intra or extrahepatic recurrence. In 2023, a computed tomography (CT) scan revealed a 1.4 cm by 1.1 cm mass in the maxillary sinus, without discernible invasion of the alveolar sinus, nasal cavity, orbital cavity, or infratemporal space. We proceeded with the resection of the maxillary sinus mass. Patho-histochemical analysis indicated that the tumor cells in the maxillary sinus were metastatic HCC cells. As of now, the patient remains in good condition with no signs of tumor recurrence.

**Conclusion:**

For patients presenting with solitary maxillary sinus metastasis, optimal liver function, and a favorable performance score, tumor resection may be the preferred treatment option. However, given the rarity of such cases, larger prospective trials are essential to determine an optimal treatment strategy that offers therapeutic benefits.

## Introduction

Primary liver cancer is the sixth most common cancer and the third greatest cause of cancer-related fatalities globally (1). Hepatocellular carcinoma (HCC) and intrahepatic cholangiocarcinoma (iCCA) are the two main kinds of primary liver cancer, with HCC being the most common (2). HCC has been demonstrated to be extremely aggressive, with most patients having a bad prognosis (1). Although there are various therapies available for HCC, curative liver resection is the most efficacious, with a 5-year overall survival rate of 60-80%. However, HCC recurrence is the most difficult therapeutic issue, with a recurrence rate ranging from 40% to 50% within 5 years following resection (3). Intrahepatic recurrence (IHR) has been proven to account for around 66% of recurrent cases, with extrahepatic recurrence (EHR) accounting for approximately 33% (4). Extrahepatic metastasis is typically indicative of a bad prognosis. Lungs, bones, lymph nodes, adrenal glands, and the brain are the most common sites of extrahepatic metastasis (5). Although cases of metastasis to the oral-facial region have been reported, metastasis to the maxillary sinus is extremely rare, with only a handful of cases reported to our knowledge (**Table 1**). Most of these cases presented with a chief complaint of epistaxis, bleeding after tooth extraction and had a liver mass present on CT scan. The various cases of HCC metastasis to the oral-facial region recorded in literature presented with concurrent liver lesions, hence since no case report of a patient with a solitary metastatic Hepatocellular carcinoma of the maxillary sinus has been documented to date, we present this extremely rare case in accordance to the CARE guidelines (11). We believe this case study presents a different phenomenon in which HCC metastasizes extrahepatically, as well as highlight a different clinical avenue to treat patients with HCC extrahepatic metastasis.

**Table 1:** Reported cases of HCC metastasis to the maxillary sinus.

Author	Age/Gender	Chief complaint	Metastasis site	Liver mass	Other metastasis	Risk factors	Treatment
**Okada et al (** 5)	67/M	Gingival bleeding	Maxillary sinus	☑	Adrenal, spleen, vertebrae, stemum	Hepatitis C	Died because of hemorrhagic shock before treatment
**Kolarević et al (** 6)	70/M	Gingival bleeding	Maxillary sinus + alveolar sinus, nasal cavity and partially infratemporal	☑	Spleen	Unknown	Chemotherapy
**Izquierdo et al (** 7)	59/M	Left epistaxis	Maxillary sinus	☑	Unknown	Hepatitis C	Unknown; died due to liver failure
**H. H. Huang et al. (** 8)	42/M	Epistaxis	Maxillary sinus	☑	Lung, clavicle	Hepatitis B	None, the patient died 3 months after the diagnosis
**Matsuda et al (** 9)	71/M	Epistaxis	Maxillary sinus	☑	Lung	Liver cirrhosis (non-B, no-C)	radiation therapy
**English et al (** 10)	44/M	Epistaxis	Maxillary sinus	x	Unknown	Unknown	Chemotherapy
**Current case**	85/M	Right maxillary sinus mass	Maxillary sinus	x	None	Liver cirrhosis (non-B, no-C)	Maxillary sinus tumor resection

☑ = present; x = absent; Patient for case by English et al. had a liver transplant.

## Presentation of case

**Figure 1:**
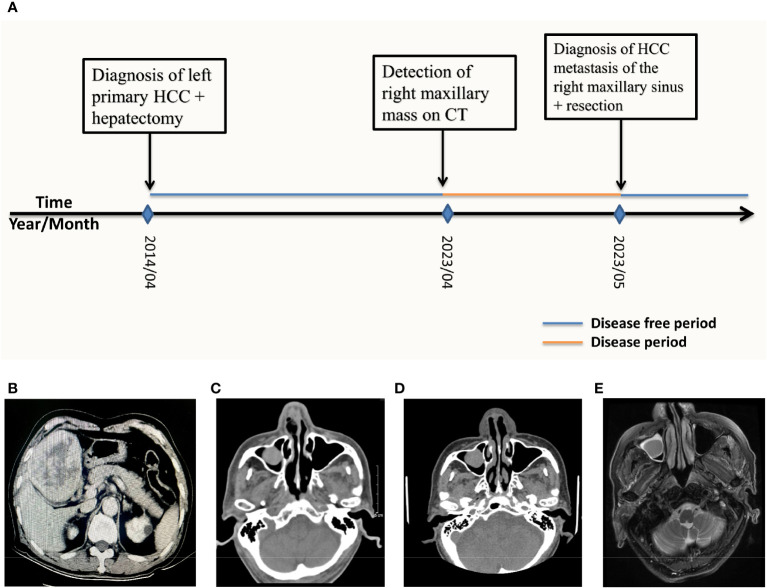
**(A)** Patient history timeline. CT scan and MRI imaging over the course of treatment. **(B)** 2014/04 Axial CT scan of the liver showing a low-density mass with dimensions 7.9cm by 9.3cm in the left inner lobe of the liver **(C)** 2023/04 CT of the head performed at another hospital showing a 1.4cm by 1.1cm mass occupying the right maxillary. **(D)** 2023/05 CT of the head obtained on admission revealed a 1.3cm by 1.2cm mass of the right maxillary sinus. **(E)** 2023/05 T2WI MRI of the head obtained on admission showed a high density 1.2cm by 0.9 cm mass in the right maxillary sinus.

0n May 19th 2023, an 85-year-old male patient was admitted to our hospital because of a mass in the right maxillary sinus. The patient had been well 1 month before admission until a surveillance head computerized tomography (CT) performed at another hospital revealed a mass 1.4 cm by 1.1 cm in diameter in the right maxillary sinus (**Figure 1C**). Upon admission, the patient complained of occasional trouble breathing from the right nostril especially after exertion, a feeling of stuffiness from the right nostril as well as blood tinged mucus from the right nostril upon heavy sneezing but he denied experiencing runny nose, headaches, eye pain or vision impairment.

**Figure 2:**
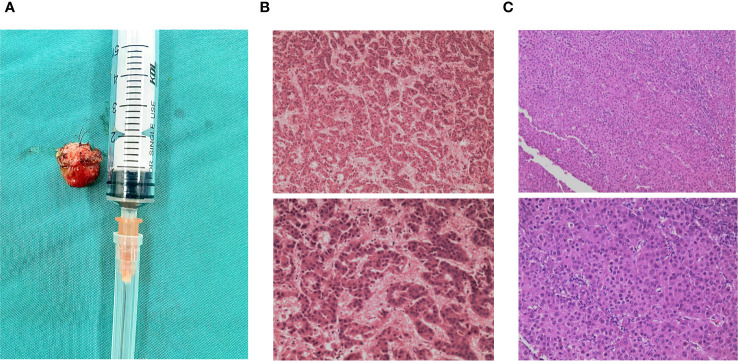
Resected maxillary sinus specimen and pathological examinations. **(A)** Specimen of the resected right maxillary sinus mass. **(B)** 2014/04: Histopathological examination of the liver mass post-resection revealed moderately differentiated hepatocellular carcinoma cells (H&E). **(C)** 2023/05: Histologic section of the right maxillary sinus lesion revealing neoplastic cells arranged in a thick trabecular pattern, consistent with metastatic hepatocellular carcinoma (H&E stain).

The patient’s prior history revealed that 9 years before admission in 2014 the patient presented to our hospital’s hepatobiliary department with a mass found in the left liver lobe on CT of the abdomen, the mass measured 9 cm by 7.5 cm in diameter (**Figure 1B**). A preliminary diagnosis of liver cancer and liver cirrhosis was made. Preoperatively the patient was classified into Child–Turcotte–Pugh class A (Child A), with a score of 5 points and was found eligible for surgery. The patient was taken for surgery where he received left curative hepatectomy. Negative resection (R0) was achieved and no complications occurred during the surgery (**Figure 2B**). After resection, the specimen was extracted from the lower abdomen in a specimen bag. The resected specimen was then sent for post-operation routine histopathological analysis which revealed that the specimen had moderately differentiated hepatocellular carcinoma cells with necrosis. A definitive diagnosis of primary HCC was made for the patient. The patient was annually followed up post hepatectomy for 9 years with no evidence of any metastasis detected on CT, magnetic resonance imaging (MRI), positron emission tomography scan (PET CT) as well as laboratory examinations performed during this period.

In conjunction with the patient’s chief complaint, the patient was admitted to our hospital’s otorhinolaryngology department. The patient’s past history was remarkable for a 20-year history of hypertension but had no history of hepatitis B virus (HBV), tuberculosis (TB), heart disease or any chronic disease. The patient denied any history of smoking, drinking alcohol and abuse of intravenous drugs. The patient was a Chinese native, married with three children, two boys and one girl who were all well. His father and mother were both deceased although the cause of death wasn’t revealed and his two siblings had no known disease.

On physical examination, the patient was slender without any signs of malnutrition and without jaundice. Abdominal examination and neurological examination were unremarkable. No changes in vision and smell were observed. All vital signs were within normal range. His performance status was 1, on a scale from 0 to 5 (with 0 indicating no adverse effects on his daily activity from the disease and 5 indicating death). A review of all systems was unremarkable.

### Timeline of the patients illness to date

This can be seen in **Figure 1A**.

### Diagnostic assessment

His laboratory examination test results revealed that his white blood count (WBC) and the serum levels of glucose, electrolytes, albumin, globulin, bilirubin and coagulation tests were all within the normal range however, his hemoglobin levels were low and renal function tests were abnormal. Alanine aminotransferase (ALT) level was 15 U/L (units per liter) and that of aspartate aminotransferase was 21 U/L, both within the normal range. The level of carcinoembryonic antigen (CEA) was 3.7 ng/L (nanograms per liter) and the alpha-fetoprotein (AFP) level was 2.6 U/L (**Table 2**).

**Table 2:** Laboratory test results on admission.

Variable	On Admission	Reference Range
Hemoglobin (g/L)	100	130-175
Hematocrit (L/L)	0.298	0.400-0.500
White Blood cell count (x10^9/L)	5.27	3.50-9.50
Neutrophils (x10^9/L)	3.62	1.80-6.30
Monocytes (x10^9/L)	0.33	0.10-0.60
Basophils (x10^9/L)	0.04	0.00-0.06
Lymphocytes (x10^9/L)	1.15	1.10-3.20
Platelet count (x10^9)	173	123-350
Total bilirubin (Umol/L)	9	0-20
Direct bilirubin (Umol/L)	<2	0-6.8
Albumin (g/L)	42.9	40-55
Sodium (mmol/L)	141	135-145
Potassium (mmol/L)	3.34	3.5-4.5
Chloride (mmol/L)	115	98-108
Urea nitrogen (mmol/L)	12.6	2.8-7.2
Creatinine (Umol/L)	266	44-97
Glucose (mmol/L)	6.8	3.9-6.1
Alanine aminotransferase (U/L)	15	9.0-50
Aspartate aminotransferase (U/L)	21	15-40
Alkaline phosphatase (U/L)	70	45-125
Prothrombin time (sec)	16.2	14-20
APTT	0.92	0.80-120
D-Dimer (mg/L)	0.42	0.00-0.50
AFP (U g/L)	2.6	0.0-13.6

Upon admission, a CT scan of the orbital and maxillary regions revealed a 1.3 cm by 1.2 cm soft tissue mass in the right maxillary sinus (**Figure 1D**), without invasion of the alveolar sinus, nasal cavity, orbital cavity and the infratemporal space. CT scan of the abdomen was unremarkable for intrahepatic metastasis, abdominal metastasis and abdominal lymph node enlargement. Chest CT was unremarkable for any lung nodules or masses. On T2-weighted imaging (T2WI) MRI of nasal and paranasal areas the tumor showed a high signal intensity mass measuring 1.2 cm by 0.9 cm with clear boundaries (**Figure 1E**). The mucous membranes of maxillary sinuses, ethmoid sinuses and sphenoid sinuses were thickened on both sides. Cystic lesions were observed in the right maxillary sinuses. The size and shape of paranasal sinuses were as usual, with good aeration, clear sinus cavity, no thick mucosa, no fluid accumulation in the cavity, and a complete bone wall. The size and shape of the nasopharyngeal cavity were good, both the eustachian tubes and the pharyngeal recess were described, and both parapharyngeal Spaces existed.

### Therapeutic interventions

After examining the imaging scans and reports as well as the patient’s willingness to resect the tumor, the team deemed the tumor resectable. After discussing the treatment plan with the patient and having obtained consent, surgery was chosen as the course of treatment. After the pre-operation evaluation was complete the patient received right maxillary sinus tumor resection under general anesthesia. Intraoperative evaluation of the tumor mass described it as a 1.5 cm by 1.5 cm, immovable smooth surfaced tumor with right nasal septum cartilage invasion. Complete resection of the tumor was achieved together with a portion of the right nasal septum cartilage. After resection, the specimen was sent for pathology (**Figure 2A**). Pathology examination revealed that the specimen was metastatic hepatocellular carcinoma (**Figure 2C**). Post-surgery histopathological examination showed a malignant metastatic tumor most consistent with that of hepatocellular carcinoma metastasis.

### Confirmed diagnosis

Solitary HCC- recurrence in the right maxillary sinus after curative resection.

### Follow up and intervention outcome

From post-operation to date, the patient is alive, and no new metastasis or recurrences have been detected during follow-ups.

## Discussion of management

Malignant tumors in the oromaxillofacial area are mostly primary; metastatic tumors account for around 1% of all oral lesions (12). The majority of cases documented with HCC metastasis to the maxillary sinus were in the 50 to 70-year age range, with an average age of 55.4 years, and were predominantly male. Okada et al. proposed that this age and gender predilection seems to be based on that of primary HCC, which occurs most frequently in men aged 45 to 60 years and takes several years to metastasize to the sinonasal regions (5). Though our patient was male which is consistent with the gender predilection of primary HCC, he was older than the previously suggested age range by 15 years.

Most metastatic tumors to the sinonasal regions originate from the lungs, kidneys, skin, and breasts. Metastasis from the liver is unusual. Cases of HCC metastasis to the upper jaw have been documented often in the literature; however, metastasis to the maxillary sinus is extremely rare (6). When HCC recurs, and especially when it manifests as metastasis to rare locations such as the maxillary sinus, it presents a scenario where the primary treatment has been exhausted, and second-line treatment strategies become crucial. Yang et al. classified extrahepatic metastases following liver resection into three types. Pattern I: first recurrence in the liver, followed by extrahepatic recurrences after multiple intrahepatic recurrences and locoregional treatments, pattern II: intrahepatic and extrahepatic recurrences exist concurrently, and pattern III: extrahepatic recurrence minus intrahepatic lesions at first recurrence (13). According to this classification, we classified our case into pattern III.

HCC extrahepatic metastasis most frequently spreads through the hematogenous, lymphatic and direct route. Metastatic HCC to the oromaxillofacial regions is generally believed to occur via the lung (6, 14). However, in our case, the patient did not have lung metastases by diagnostic imaging. It has been postulated that in the absence of lung metastases, metastases might arise via the vertebral and azygos vein systems or the lymphatic system. In the case of liver cirrhosis, the former method is preferred (5, 15, 16). Since our case had liver cirrhosis, we postulate that metastasis by the vertebral vein system was more likely.

The diagnosis of metastatic tumors in the oromaxillofacial area might be difficult, especially when the underlying tumor has not yet been identified. Patients most frequently present to the hospital complaining of epistaxis however, depending on the location of the tumor other less common symptoms included diplopia, gingival hemorrhage, nasal blockage, proptosis, headache, and cheek pain. However, these symptoms and manifestations are the same as those that may be caused by primary head and neck tumors, and there are no distinguishing clinical or radiologic characteristics that may quickly distinguish primary from metastatic lesions (8). If a patient has a history of HCC or another primary tumor, for instance, that history may be the only indicator. By analyzing previously reported cases, Pires et al. revealed that metastatic oromaxillofacial tumors were detected before the initial hepatic lesion in 66% of patients (17). In our case, although the patient presented with a painless, non-bleeding tumor detected on head CT, his past diagnosis of primary HCC and histopathological results post hepatectomy supporting that diagnosis 9 years prior to admission gave us clues to the possibility of the patient having HCC metastasis.

### Pathological examination discussion

Pathological examination plays a vital role in diagnosing extrahepatic metastasis. HepPar-1 antibody is regarded as being one of the most specific and sensitive indicators for HCC (6). Additionally, in metastatic hepatocellular cancer cells, cytokeratin 7 (CK-7) is nearly almost always negative, which distinguishes them from other adenocarcinoma cells. The acquisition of medical history is crucial in the diagnosis of non-primary tumors. HepPar-1, AFP, GPC3, and Arg-1 are four biomarkers that are vital in the diagnosis of metastatic hepatocellular carcinoma (18).

In our case pathological examination of the resected specimen from the maxillary sinus showed hepatocellular carcinoma at low power demonstrating trabecular morphology, tumor cells appeared larger, had more hyperchromatic nuclei with less acidophilic cytoplasm, and had marked nuclear atypia at high power in hematoxylin and eosin stain (HE stain). Tumor cells showed strong immunoreactive staining for HepPar-1 and were positive for CD-10, GPC-3, and CK but negative for CK-7 and Arginase-1.

### Surgical treatment discussion

In most HCC cases surgical excision is the first-line treatment. However, patients with advanced HCC or compromised liver function often face a challenging prognosis, necessitating second-line treatment options when surgical intervention is unfeasible (19).

There is no unanimity on which second-line treatment to use for advanced stage patients thus challenges exist for clinicians especially when it comes decision making. When surgical excision is not feasible, anticancer agents are presently used as second-line treatment options (19). Many new target drugs for advanced HCC continue to emerge from clinical trials, however, despite these advancements in systemic therapeutic strategies, cases resistant to chemotherapy pose a formidable challenge.

Sorafenib, a multi-target tyrosine-kinase inhibitor (TKI), was among the initial drugs to demonstrate efficacy in improving disease outcomes for advanced HCC (20) Data from both the SHARP and Asia-Pacific randomized trial more than a decade ago showed substantial evidence of an improvement in overall survival (OS) when sorafenib was compared to placebo (21, 22). Its approval by the FDA in 2007 marked a significant milestone (23). Sorafenib is currently still the conventional second-line treatment for advanced HCC, nonetheless, the overall survival (OS) for patients treated with sorafenib remains less than a year (24). In subsequent years, a number randomized control trials (RCTs) showed negative results. In a randomized phase 3 trial Kudo et al. provided insights into the comparative effectiveness of lenvatinib, showcasing its non-inferiority to sorafenib in selected patients with treatment-naive advanced HCC (25).

A pivotal development in this regard is the approved use of atezolizumab plus bevacizumab as a first-line treatment for unresectable HCC, as demonstrated in the IMbrave 150 study. This combination therapy exhibited superior outcomes in terms of OS and progression-free survival (PFS) when compared to sorafenib. Specifically, the 12-month OS rate reached 67.2% with atezolizumab–bevacizumab, surpassing the 54.6% observed with sorafenib (26).

Immunotherapy, particularly anti-PD-1/PD-L1 and anti-CTLA-4 antibodies, has shown promise as a treatment for advanced HCC in recent years (27). It has been a breakthrough in anti-cancer therapy and has completely revolutionized treatment strategies. Amongst the drugs that have shown clinical evidence in HCC is nivolumab and pembrolizumab. In a multicenter, open label phase II study, the CheckMate-040 study’s survival outcomes were found promising, having survival rates of 83% and 74% at 6 and 9 months, respectively, and a median time to progression (mTTP) of 4 months. Its 18-month overall survival rates were 57% for those who had never had sorafenib therapy and 44% for those who had. The median overall survival (mOS) for sorafenib-naive patients increased to 28.6 months and 15.6 months for sorafenib-experienced patients (28). Pembrolizumab was assessed the KEYNOTE-224 nonrandomized multicenter phase II trial and it proved to have a mTTP of 4.9 months and a mOS of 12.9 months (29).

When extrahepatic metastasis occurs, the prognosis of surgical resection is frequently poor (4, 30). Only patients with Child class B or better liver function and an Eastern Cooperative Oncology Group (ECOG) performance status 0-2 are usually considered for extrahepatic metastatic lesions resections. According to Uchino et al., the 1-year survival rate was 39.3%, while the 3-year and 5-year overall survival (OS) rates were 7.4% and 4%, respectively (31). In cases of extrahepatic metastasis, an examination of the causes of death indicated that many deaths were due to liver failure caused by the degradation of liver cirrhosis or intrahepatic cancer progression (32). Therefore, while considering whether or not to treat extrahepatic metastasis, it is critical to examine the degree of advancement of both the extrahepatic lesion and any intrahepatic lesions, as well as the degree of hepatic function.

Resection may be suggested for patients with solitary metastases in correspondence to the Chinese National Liver Cancer staging classification and the consensus on multidisciplinary care of recurrent and metastatic HCC following resection (33). Few case reports address the efficacy of surgical resection for extrahepatic metastases. According to Lam et al., resection was effective in 9 cases of lung metastasis, while Taniai et al. and Rousselet et al. documented multiple successful cases of adrenal metastatic resection (34, 35). However, no case has been documented showing the efficacy of resection in maxillary sinus metastasis patients. Most of the documented cases of HCC extrahepatic metastasis to the maxillary sinus received chemotherapy and radiotherapy as the first line of treatment (**Table 1**).

The patient described in this paper had no liver mass and no metastases discovered in any portion of the body, including lymph nodes. Although liver cirrhosis was observed, viral hepatitis infection B (HBV) was negative. Bilirubin, albumin, transaminases, alkaline phosphatase, and alpha-fetoprotein levels were all within normal limits, other laboratory tests are shown in (**Table 2**). ECOG’s (Eastern Cooperative Oncology Group) performance status was 1. The tumor margins were smooth with a partial invasion of the nasal cartilage. After preoperative evaluation and after consultation with the multidisciplinary team, surgery was chosen as the option of treatment to excise the maxillary sinus tumor since the patient retained good liver function, a good performance score and a solitary extrahepatic metastatic mass. The surgery was uneventful and R0 resection was achieved. The patient is still alive and continues to come to our outpatient department for regular follow-ups.

In summary, HCC metastases to the maxillary sinus are extremely aggressive and rare. Of the few recorded cases so far, this is the only documented case that had a solitary right maxillary sinus HCC metastatic tumor and the only case that received surgery to resect the tumor. Immunohistochemistry results with hepatocytes, elevated AFP levels and primary liver cancer, are valuable diagnostic signs.

## Case limitations and suggestions for future research

Our study relies on a single successful case at a single center, which restricts the generalizability of the findings. We believe larger multicenter collaborative cohort studies can be conducted to enhance the robustness of conclusions and facilitate a more comprehensive understanding of HCC maxillary sinus metastasis. This case lacks a comparative analysis between surgical intervention and alternative treatments, such as systemic chemotherapy. Future comparative studies would aid in determining the most effective treatment strategy, considering both surgical and non-surgical interventions for HCC extrahepatic metastasis. Our study doesn’t provide long-term outcome data beyond the immediate post-operative period. In the future investigations should include extended follow-up periods to assess the durability of treatment outcomes, recurrence rates, and overall survival, providing a more comprehensive perspective on the efficacy of surgical intervention. The study does not delve into potential variations in patient response to surgical intervention, considering factors like age, comorbidities, and overall health status. Future research should incorporate a more detailed analysis of patient characteristics and factors influencing treatment outcomes, providing a nuanced understanding of the feasibility and effectiveness of surgery in diverse patient populations. This case study highlights the rarity of HCC maxillary sinus metastasis but falls short of establishing optimal treatment guidelines. Research efforts should focus on developing standardized protocols for the management of HCC extrahepatic metastasis, considering factors like tumor characteristics, patient health, and treatment preferences. Addressing these limitations will contribute to a more comprehensive and evidence-based approach to the management of HCC maxillary sinus metastasis, guiding clinicians in making informed treatment decisions.

## Patient perspective

We contacted the patient after the surgery and before writing this paper asking him of his views on our therapy. The patient neither had complaints nor questions. Post operation to date he has no symptoms to complain. He thought the treatment was a success.

## Data availability statement

The original contributions presented in the study are included in the article/supplementary material. Further inquiries can be directed to the corresponding author.

## Ethics statement

The studies involving humans were approved by Ethics committee of The First Affiliated Hospital of Wenzhou Medical University. The studies were conducted in accordance with the local legislation and institutional requirements. The participants provided their written informed consent to participate in this study. Written informed consent was obtained from the individual(s) for the publication of any potentially identifiable images or data included in this article.

## Author contributions

TM: Conceptualization, Data curation, Investigation, Visualization, Writing – original draft, Writing – review & editing. YS: Conceptualization, Data curation, Formal analysis, Funding acquisition, Investigation, Methodology, Resources, Supervision, Validation, Visualization, Writing – original draft, Writing – review & editing.
